# Tri-Magnesium Phosphate as a Candidate Biocompatible Retarder for Magnesium Potassium Phosphate Cement: Setting Behavior, Mechanical Properties, and Microstructure

**DOI:** 10.3390/ma19071354

**Published:** 2026-03-29

**Authors:** Yuanquan Yang, Xiaoyu Ying, Hao Huang, Yunpeng Cui

**Affiliations:** 1 School of Architecture and Civil Engineering, Liuzhou Institute of Technology, Liuzhou 545616, China; 2 School of Materials Science and Engineering, Shenyang Ligong University, Shenyang 110159, China

**Keywords:** magnesium potassium phosphate cement (MKPC), potential biocompatibility, retarder, trimagnesium phosphate (TMP)

## Abstract

Magnesium potassium phosphate cement (MKPC) is a promising bone repair material but suffers from excessively rapid setting time (typically within minutes) that limits clinical application. This study systematically investigates trimagnesium phosphate (TMP) as a candidate retarding additive for MKPC. TMP was used to partially replace dead-burned magnesium oxide at replacement levels of 0%, 10%, and 15% by mass. The effects of TMP content, water-to-cement ratio (0.17–0.23), and magnesium-to-phosphate molar ratio (4–10) on setting time, fluidity, hydration kinetics, compressive strength, and microstructure were comprehensively evaluated. Results show that TMP effectively extends the setting time from 9–13 min (without TMP) to 10–19 min, providing a working window that may be suitable for biomedical applications requiring extended handling time. Notably, 10% TMP incorporation enhances early compressive strength, with 1-day strength reaching 35.2 MPa compared to 28.5 MPa for control samples. Hydration heat analysis reveals TMP moderates the acid-base reaction kinetics through its slower dissolution rate compared to MgO. Microstructural characterization shows TMP promotes the formation of denser K-struvite crystals with refined microstructure. The optimal TMP dosage of 10% achieves a balanced performance, extending setting time while improving early strength and microstructural densification. These findings establish TMP as an effective retarder for developing MKPC-based materials with potential for biomedical applications, pending further biological validation.

## 1. Introduction

Bone injuries, defects, and degenerative conditions pose significant challenges in the field of orthopedics and regenerative medicine [1,2]. Traditional treatment methods, such as autografts and allografts, are often limited by availability, donor site morbidity, and the risk of immune rejection [3,4]. In this context, the emergence of magnesium potassium phosphate cement (MKPC) has garnered substantial attention as a promising alternative for bone repair and regeneration [5,6,7]. MKPC is an emerging class of inorganic cements that have garnered significant attention in the field of bone repair and regeneration [8,9,10]. Unlike traditional calcium-based cements [11,12], MKPC offers a unique set of properties that make it a promising candidate for various orthopedic applications [13,14,15]. MKPC is non-toxic, non-inflammatory, and does not elicit adverse immune responses when implanted in the body. Additionally, MKPC exhibits a neutral pH during the setting process [16,17], which is crucial for maintaining the viability of surrounding cells and tissues. In addition, MKPC exhibits high compressive strength [18,19], which can reach up to 50 MPa, making it comparable to the strength of human cortical bone. This high mechanical strength suggests potential for load-bearing applications, such as the repair of fractures or the reinforcement of weakened bone structures, although such applications require further validation. MKPC is composed of magnesium oxide (MgO), potassium phosphate (KH_2_PO_4_), and water [7,20,21]. As the components are mixed, they undergo a rapid acid-base reaction, resulting in the formation of magnesium potassium phosphate hexahydrate (MgKPO_4_·6H_2_O) (Equation (1)), also known as K-struvite [22,23,24]. Currently, calcium phosphate cements (CPCs) are the most widely used inorganic bone repair materials in clinical practice due to their excellent biocompatibility and osteoconductivity [11,12]. However, CPCs generally exhibit low mechanical strength (typically <10 MPa) and relatively slow setting, which limit their use in load-bearing applications. In contrast, MKPC offers superior mechanical properties and rapid setting, making it a promising alternative for applications requiring early strength development [5,6,7].

(1) $$\mathrm{MgO}+{\mathrm{KH}}_{2}{\mathrm{PO}}_{4}+5H_{2}O\to {\mathrm{MgKPO}}_{4}\cdot 6H_{2}O$$

However, the intense reaction kinetics of MKPC lead to excessively rapid setting (typically within minutes), posing significant challenges for clinical surgical procedures [25]. The clinical significance of controlled setting is paramount for bone repair applications, where surgeons require adequate working time for precise placement and shaping of the cement. Excessive setting acceleration can lead to incomplete filling of bone defects and compromised interfacial bonding. Simultaneously, the concentrated hydration heat released during the reaction can easily induce microcracks within the material, severely limiting its biomedical applications. Therefore, the introduction of retarders to regulate its hydration process represents a key technical pathway for enhancing its workability and application potential [26]. Current research on MKPC retarders has developed into a diversified system, primarily including: (1) Boron-based compounds, such as borax and boric acid, which are the most classic and effective retarders. They form a dense magnesium borate coating (e.g., Mg_3_B_2_(PO_4_)_2_(OH)_6_·6H_2_O) on the surface of active MgO particles, physically blocking contact between reactants [23]; (2) Phosphates, such as sodium tripolyphosphate, which adsorb onto the surface of hydration product nuclei and chelate Mg^2+^ in solution, effectively delaying the nucleation and growth of crystalline phases such as struvite [27]; (3) Organic acids and biopolymers, such as citric acid and sodium alginate, which reduce the concentration of free Mg^2+^ in solution through complexation, while also retarding crystal precipitation via steric hindrance [27]. Through these distinct physicochemical mechanisms, these retarders have successfully extended the setting time of MKPC from minutes to tens of minutes or even hours, significantly improving its workability. Despite notable progress in retarder research, several critical challenges remain in practical application. Firstly, the trade-off between properties is prominent: most retarders inevitably compromise early-age strength and its development rate while prolonging setting time; excessive dosage can even lead to a loose structure, impairing the final mechanical performance and durability of the material [5,27]. Secondly, limitations in applicability and cost-effectiveness exist: for example, boron-based retarders exhibit reduced effectiveness at elevated temperatures and may fail in the presence of calcium-containing admixtures (e.g., slag) due to the formation of CaB_4_O_7_; meanwhile, some highly effective organic retarders are expensive. More importantly, with the growing exploration of MPC in biomedical fields such as bone repair materials, unprecedented new demands have been placed on retarders. Traditional retarders like borax are no longer suitable due to potential cytotoxicity; instead, retarders must now possess excellent biocompatibility and degradability. Furthermore, the setting time must be precisely controlled within a narrow window that not only meets surgical handling requirements (typically 10–30 min) but also allows rapid development of initial support strength—all without compromising the material’s inherent bioactivity and osseointegration capacity. In the present study, we aim to extend the setting time of MKPC from its original range of 9–13 min into this target window (10–30 min) by incorporating trimagnesium phosphate (TMP) as a candidate retarder, while maintaining or even enhancing mechanical performance. Trimagnesium phosphate (TMP, Mg_3_ (PO_4_)_2_) is an inorganic compound composed of magnesium and phosphate ions, which are essential elements in bone tissue [28,29,30]. It has been reported to exhibit favorable biocompatibility and to promote hydroxyapatite formation, making it a promising candidate for biomedical applications [31]. Compared to highly reactive dead-burned MgO, TMP has a slower dissolution rate, which may moderate the acid-base reaction kinetics of MKPC and potentially serve as a “reactive retarder” that simultaneously controls setting behavior and contributes to matrix densification.

This study aims to systematically investigate the effects of trimagnesium phosphate (TMP) as a candidate retarder/reactive magnesium source on the properties and hydration process of magnesium potassium phosphate cement (MKPC). This study addresses three key scientific questions: (1) Can TMP effectively regulate MKPC setting without sacrificing mechanical performance? (2) What is the optimal TMP dosage that balances setting control and strength development? (3) How does TMP’s dual role as retarder and reactive component influence the hydration mechanism and microstructure evolution? Specifically, TMP was used to partially replace MgO, and its influence on the setting time, fluidity, hydration heat release, compressive strength, hydration products, and microstructure of MKPC was comprehensively evaluated. The underlying mechanism of TMP was also elucidated. This work is expected to provide new insights and experimental evidence for the development of high-performance and biocompatible MKPC-based bone repair materials.

## 2. Experimental Section

### 2.1. Materials

The magnesium potassium phosphate cement (MKPC) pastes in this study were formulated using the following raw materials: dead-burned magnesium oxide (MgO, calcined at 1600 °C, Liaoning Jinding Magnesite Group Co., Ltd., Anshan, China), trimagnesium phosphate (TMP, Mg_3_(PO_4_)_2_), potassium dihydrogen phosphate (KH_2_PO_4_, KDP), and distilled water. The KDP and TMP, both of analytical grade, were supplied by Sinopharm Chemical Reagent Co., Ltd., Shanghai, China. The MgO powder had a chemical purity of 98.9% and a specific surface area of 1124 m^2^/kg, as measured by the Brunauer–Emmett–Teller (BET) method. The particle size distribution of MgO was determined by laser diffraction, with a surface-area-weighted mean diameter D[3,2] of 4.85 μm.

### 2.2. Mix Design

The mix proportion for MKPC pastes is shown in Table 1. To investigate the impact of TMP on the hydration properties of MKPC, the magnesium oxide to potassium dihydrogen phosphate ratio (M/P) by molar was set as 4, 6, 8 and 10, respectively. The mass ratio of water/cement (W/C) was set as 0.17, 0.19, 0.21 and 0.23. The calcined magnesium oxide was partially replaced by TMP in quantities of 0, 10% and 15%.

The selected W/C and M/P ratios were based on previous studies on MKPC systems [16] and preliminary experiments conducted in our laboratory. The M/P ratios of 4, 6, 8, and 10 cover the typical range reported in the literature, where lower M/P ratios (4–6) are associated with sufficient phosphate for complete hydration, while higher ratios (8–10) represent excess MgO that can act as micro-aggregates but may also lead to incomplete reaction [32]. The W/C ratios of 0.17 to 0.23 were chosen to encompass the range commonly used for MKPC pastes, where lower W/C ratios (0.17–0.19) favor higher mechanical strength but may reduce workability, and higher ratios (0.21–0.23) improve fluidity but may dilute the reaction system [33]. These ranges allow a systematic evaluation of TMP effects under varying mix proportion conditions.

The experimental design followed a sequential approach, where each series was designed to isolate the effect of one variable while keeping the others constant. Three variables were systematically investigated: TMP content (0%, 10%, 15%), water-to-cement (W/C) ratio (0.17, 0.19, 0.21, 0.23), and magnesium-to-phosphate (M/P) molar ratio (4, 6, 8, 10). A total of 9 distinct mixtures were prepared, as summarized in Table 1. The reference mixture (0% TMP, M/P = 4, W/C = 0.21) served as the baseline for all comparisons.

The pastes were prepared by dry-mixing the solid powders (MgO, TMP, KDP) for 2 min in a planetary mixer to ensure homogeneity, followed by the addition of distilled water and mixing for an additional 3 min. The fresh paste was then cast into 20 mm × 20 mm × 20 mm steel molds. The molds were covered with plastic films to prevent moisture evaporation and cured at a constant temperature of 25 ± 2 °C and a relative humidity (RH) of 50 ± 5% for 2 h. After demolding, the specimens were continuously cured under the same environmental conditions (25 °C, 50% RH) until the designated testing ages of 1 day and 7 days.

### 2.3. Test Methods

The setting time was measured according to Chinese standard JGJ/T70-2009 (Standard for test method of basic properties of construction mortar) [34]. The time between the initial contact of the sample with water and the attainment of a penetration resistance value exceeding 0.5 mm was recorded. The resulting value is accurate to within 30 s.

The fluidity was determined in accordance with Chinese standard GB/T 2419 [35]. During the test, the truncated cone mould is to be lifted vertically upwards, allowing the mixture to flow freely (without opening the table jumping) for a period of 30 s. Thereafter, the diameters of two perpendicular directions on the bottom surface of the mixture are to be measured with calipers, and the average value calculated. The integer value in millimetres is then to be taken. The arithmetic mean value represents the fluidity of the sample. The fluidity test is completed within a five-minute period.

Isothermal conduction calorimetry was carried out using a TAM-air (eight-channel calorimeter, TA instrument, New Castle, DE, USA) instrument. For each group, 1 g of the cement and corresponding water (according to Table 1) were placed in a 20 mL ampoule and a 1 mL syringe, respectively. Then the ampoule and syringe were placed in a TAM-air apparatus at 20 °C. The solution from the syringe was injected into the ampoule and stirred for 2 min with the inbuilt stirrer after the instrument was stabilized, while data collection was started.

The compressive strength tests were conducted according to Chinese standard JC/T2537-2019 [36]. The results were the average strength values from 6 samples in each group. After completing the mechanical strength tests, the test specimens were soaked in anhydrous ethanol for XRD, FTIR, TG/DSC and microscopic testing. Phase identification was performed using an Ultima IV X-ray diffractometer (Rigaku, Tokyo, Japan) with Cu-Kα radiation (λ = 1.5418 Å) operated at 40 kV and 40 mA. Data were collected in the 2θ range of 5° to 80° with a step size of 0.02°. Quantitative phase analysis was conducted using the Rietveld refinement method. The TG/DSC test was carried out using a Netzsch STA 449 F3 analyser (NETZSCH Analyzing & Testing, Selb, Germany) at a heating rate of 10 °C/min from 30 °C to 500 °C. Chemical bonding information was obtained using a Thermo Fisher Nicolet iS50 spectrometer (Thermo Fisher Scientific, Waltham, MA, USA) in the attenuated total reflection (ATR) mode. Spectra were recorded in the wavenumber range of 4000 to 400 cm^−1^ with a resolution of 4 cm^−1^. The microstructure and morphology of fracture surfaces from selected samples were observed using a TESCAN Maia3 field-emission scanning electron microscope (TESCAN, Brno, Czech Republic). Prior to observation, the samples were sputter-coated with a thin layer of gold to enhance conductivity.

All experiments were performed in triplicate for setting time and fluidity measurements, and six specimens were tested for compressive strength at each curing age. Results are expressed as mean ± standard deviation (SD). Statistical analysis was performed using one-way analysis of variance (ANOVA) followed by Tukey’s post hoc test for multiple comparisons, and Student’s *t*-test for two-group comparisons. A significance level of *p* < 0.05 was considered statistically significant. All tests were performed in accordance with the specified Chinese standards, which are widely accepted in the field of cement and construction materials research.

## 3. Results

### 3.1. Setting Time and Fluidity

**Figure 1:**
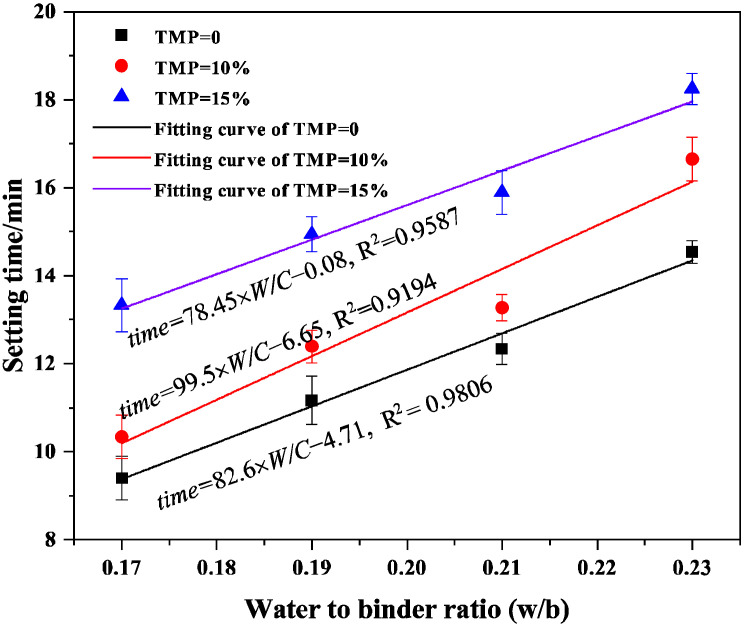
Setting time of the MKPC with TMP at variable W/C ratios (M/P = 4).

**Figure 2:**
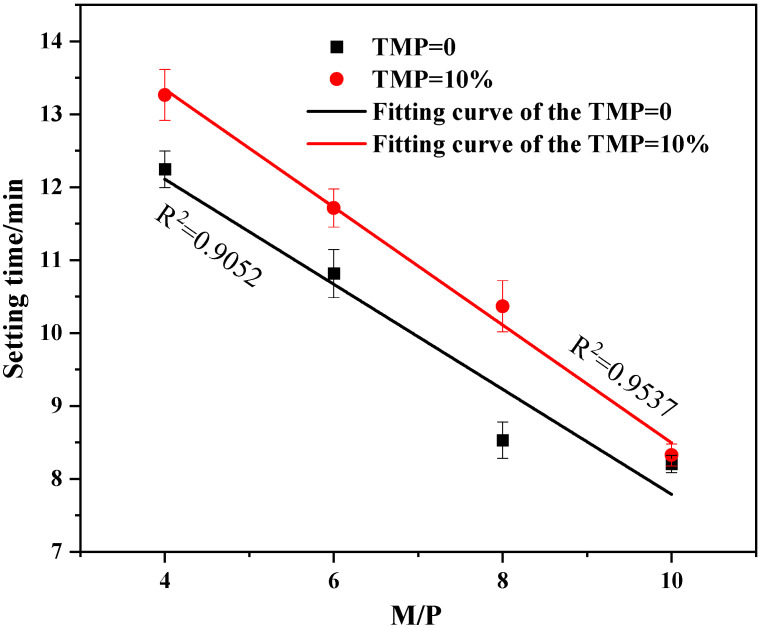
Setting time of the MKPC with TMP at variable M/P ratios (W/C = 0.21).

The influence of TMP content, W/C ratio, and M/P ratio on the setting behavior of MKPC is summarized in Figure 1 and Figure 2. As shown in Figure 1, for the reference pastes without TMP (TMP = 0%), the setting time exhibits a clear increasing trend with the elevation of the W/C ratio from 0.17 to 0.23. This observation is consistent with the findings reported by Li et al. [32], who noted that a higher water content dilutes the concentration of reacting ions and increases the inter-particle distance, thereby slowing down the dissolution-precipitation kinetics and prolonging the initial and final setting. The setting times for these reference pastes ranged from 9 to 13 min under the tested conditions.

The incorporation of TMP significantly altered the setting behavior. For pastes containing 10% and 15% TMP (by mass replacement of MgO), the setting times were effectively prolonged across all W/C ratios, extending the range to approximately 10–19 min. This confirms the retarding effect of TMP in the MKPC system. The retardation mechanism can be primarily attributed to the lower reactivity and slower dissolution rate of TMP compared to the highly reactive dead-burned MgO. When TMP partially replaces MgO, it provides a less intense and more sustained source of Mg^2+^ ions, moderating the overall acid-base reaction rate between MgO and KH_2_PO_4_. Consequently, the rapid formation and growth of the primary hydration product, K-struvite (MgKPO_4_·6H_2_O), are delayed. As shown in Figure 1, the setting time data are expressed as mean ± SD (*n* = 3). Statistical analysis confirmed that the addition of TMP significantly prolonged the setting time at all W/C ratios compared to the reference group (*p* < 0.05 for all comparisons). The retarding effect was more pronounced with higher TMP content, with the 15% TMP group exhibiting significantly longer setting times than the 10% TMP group at W/C ratios of 0.19 and 0.21 (*p* < 0.05).

Notably, as shown in Figure 2, the setting time also exhibited a strong dependence on the M/P molar ratio. For a given TMP content and W/C ratio, a higher M/P ratio (indicating a relative excess of MgO) generally led to a shorter setting time. This is because a higher MgO content provides more reactive sites and drives the reaction forward more aggressively. However, even at higher M/P ratios, the retarding effect of TMP remained evident, as pastes containing TMP consistently exhibited longer setting times compared to their TMP-free counterparts at the same M/P and W/C ratios. In summary, the setting time of MKPC is governed by a synergistic interplay of the W/C ratio, M/P ratio, and TMP content. The successful extension of the workable time window to 10–19 min through TMP incorporation is of paramount practical significance. This range is highly suitable for clinical handling and placement during surgical procedures for bone repair [1]. Combined with its inherent biocompatibility and degradability [28], TMP demonstrates strong potential as a promising candidate retarder for bio-applications of MKPC, effectively addressing the critical challenge of excessively rapid setting without introducing potentially cytotoxic chemical additives. The setting time data at variable M/P ratios (Figure 2) are presented as mean ± SD (*n* = 3). At all M/P ratios, both 10% and 15% TMP groups exhibited significantly longer setting times compared to the TMP-free group (*p* < 0.05). Notably, the retarding effect of TMP remained significant even at the highest M/P ratio of 10 (*p* < 0.05), indicating that TMP effectively counteracts the accelerating effect of excess MgO.

**Figure 3:**
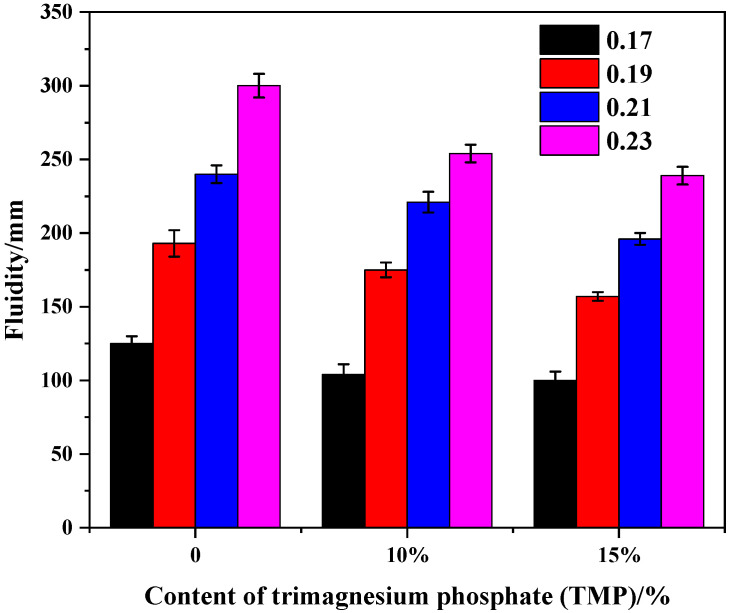
Fluidity of the MKPC with TMP at variable W/C ratios (M/P = 4).

**Figure 4:**
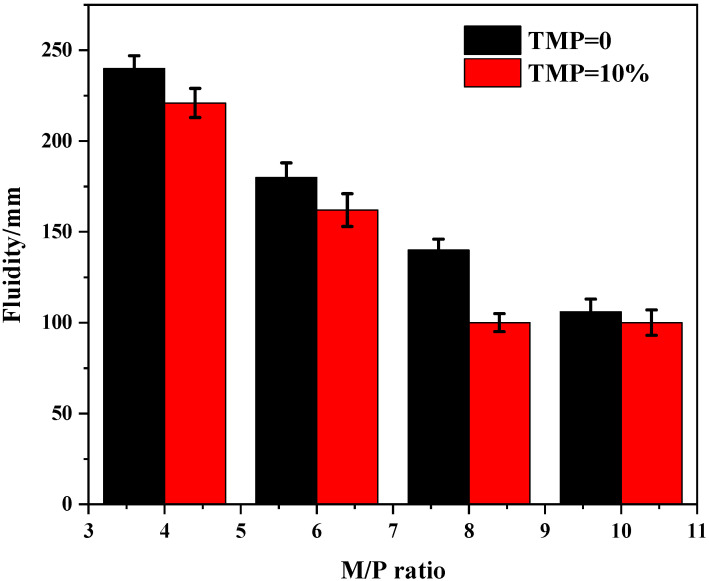
Fluidity of the MKPC with TMP at variable M/P ratios (W/C = 0.21).

Figure 3 and Figure 4 collectively analyze the influence of TMP as a partial replacement of the magnesium source on the fluidity of MKPC paste, which serves as a key indicator for evaluating whether its use as a retarder compromises the material’s workability. Overall, the incorporation of TMP only exerts a limited and controllable negative effect on the fluidity of MKPC, thereby providing a critical foundation for its practical process feasibility. Specifically, as shown in Figure 3, the water-to-cement (W/C) ratio is the predominant factor determining fluidity. Across all series, increasing the W/C ratio consistently results in a significant enhancement of fluidity. Although the addition of TMP (e.g., 10%) leads to a systematic slight reduction in fluidity compared to the reference group under the same conditions (TMP = 0%), with a decrease of approximately 25–50 mm, an appropriate adjustment of the W/C ratio can still enable TMP-containing pastes to achieve workability that meets construction requirements (e.g., reaching 250 mm). This result indicates that the incorporation of TMP does not fundamentally alter the rheological characteristics of the paste, and its slight thickening effect can be effectively compensated for through conventional mix design adjustments. Fluidity results are expressed as mean ± SD (*n* = 3), as shown in Figure 3. While the addition of TMP led to a slight reduction in fluidity at all W/C ratios, statistical analysis indicated that the difference between the 10% TMP group and the reference group was only significant at W/C = 0.21 and 0.23 (*p* < 0.05), whereas the 15% TMP group exhibited significantly lower fluidity than the reference group at all W/C ratios (*p* < 0.05).

On the other hand, Figure 4 indicates that the magnesium-to-phosphate (M/P) ratio exerts a more pronounced influence on fluidity, where an increase in the M/P ratio significantly reduces fluidity due to the higher content of reactive MgO. Notably, under this dominant influencing factor, the additional fluidity loss caused by the incorporation of TMP becomes relatively weaker, with its curve tending to parallel that of the reference group. This further suggests that in complex mix proportion systems, TMP itself is not the dominant factor controlling fluidity. Its primary mechanism as a retarder lies in extending the operational window by slowing down reaction kinetics—as demonstrated in previous analyses of setting time and hydration heat—rather than significantly altering the initial rheological state. In summary, while TMP effectively fulfills its retarding function, it exerts only a limited impact on the initial workability of MKPC paste. This characteristic enables it to meet the precise control requirements for workable time (10–30 min) in bone repair materials without severely compromising constructability, thereby strengthening its overall potential as a biocompatible retarder. The fluidity data at variable M/P ratios (Figure 4) are presented as mean ± SD (*n* = 3). The M/P ratio was identified as the dominant factor influencing fluidity, with higher M/P ratios leading to significantly reduced fluidity across all TMP contents (*p* < 0.05). The additional fluidity loss caused by TMP incorporation was relatively minor and only statistically significant for the 15% TMP group at M/P ratios of 4 and 6 (*p* < 0.05).

### 3.2. Calorimetry Analysis

**Figure 5:**
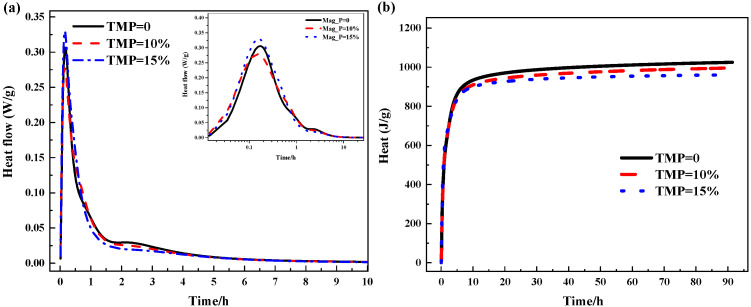
Hydration of the MKPC with TMP (W/C = 0.21 and M/P = 4): (**a**) Heat flow and (**b**) heat.

**Figure 6:**
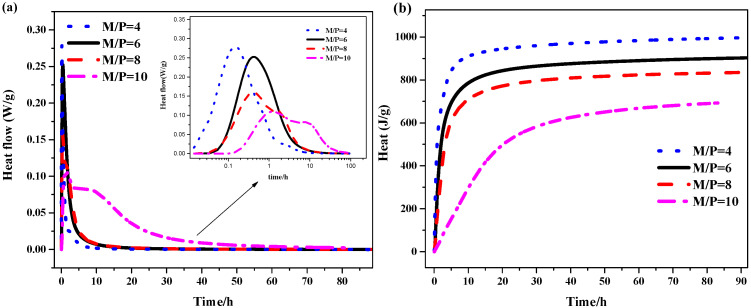
Hydration of the MKPC with TMP at variable M/P ratios (W/C = 0.21 and TMP = 10%): (**a**) Heat flow and (**b**) cumulative heat release.

Figure 5 and Figure 6 illustrate the effect of TMP on the hydration kinetics of MKPC (heat release rate and cumulative heat release over 90 h), the results of which directly explain the setting and fluidity development behaviors described earlier. The hydration heat flow curves of all samples exhibit typical characteristics of the MKPC system: an initial rapid dissolution peak (first peak), followed by a main exothermic peak corresponding to the crystallization of K-struvite (second peak). However, the introduction of TMP significantly alters the intensity and temporal distribution of these characteristic peaks.

As shown in Figure 5a, with increasing TMP content (from 0% to 15%), the intensity of the main exothermic peak (second peak) decreases significantly, and its occurrence time is delayed. This clearly confirms the retarding effect of TMP. The mechanism lies in the fact that TMP, as a magnesium source with low solubility, releases Mg^2+^ at a much slower rate compared to highly reactive MgO, thereby reducing the early supersaturation of Mg^2+^ in the solution and delaying the explosive nucleation and growth of K-struvite crystals. It is worth noting that the incorporation of TMP also slightly advances the first dissolution peak, which may be related to its initial surface wetting and dissolution behavior. However, the energy contribution of this peak is minimal and exerts far less influence on the overall hydration process than the retarding effect of the main peak. Correspondingly, Figure 5b shows that the cumulative heat release during the early stage (e.g., within the first 24 h) changes little, but the total heat release over 90 h systematically decreases with increasing TMP content. This is directly attributed to the replacement of a portion of highly exothermic MgO by low-reactivity TMP, which reduces the total amount of “fuel” available for rapid acid-base neutralization in the system. This trend is consistent with the slowed development of compressive strength at higher TMP contents (e.g., 15%).

More importantly, the introduction of TMP reverses the conventional response of the MKPC system to changes in the M/P ratio. According to Xu et al. [33], in MKPC without TMP, increasing the M/P ratio (i.e., raising the relative content of MgO) accelerates the reaction, causing the main exothermic peak to occur earlier and more intensely. However, as shown in Figure 6a, in the system containing TMP, increasing the M/P ratio instead leads to a further delay and weakening of the main exothermic peak. This seemingly contradictory phenomenon provides insight into the key mechanism of TMP action. In this study, TMP was used to replace MgO at a fixed mass percentage across all M/P ratios. Consequently, when the M/P ratio increases (i.e., the total MgO content increases relative to KH_2_PO_4_), the absolute amount of TMP also increases proportionally, while its replacement ratio remains constant. Thus, in the presence of TMP, the total magnesium source in the system consists of both rapidly reactive MgO and slowly releasing TMP. Increasing the M/P ratio means that the absolute amount of the inert/slow-release component (TMP) also increases, and its overall dilution and slow-release effects may outweigh the accelerating effect contributed by the additional reactive MgO, thus manifesting as further retardation on the macroscopic scale. This explains, from the perspective of hydration kinetics, why the setting time of TMP-containing pastes can still be effectively extended even at high M/P ratios (see Figure 2). Figure 6b shows that, regardless of TMP incorporation, the total cumulative heat release decreases with increasing M/P ratio, which aligns with the theoretical expectation of a relative deficiency of the reactant KH_2_PO_4_. This indicates that TMP does not alter the thermodynamic equilibrium endpoint of the reaction but primarily influences the kinetic pathway by which equilibrium is reached.

The hydration heat analysis demonstrates that TMP, through its slow dissolution behavior, acts as a “slow-release reservoir” for magnesium ions, effectively delaying the progression of the main hydration reaction peak in MKPC. This directly leads to the extension of setting time. Furthermore, the presence of TMP alters the system’s sensitivity to the M/P ratio, enabling it to maintain its retarding effect even at high M/P ratios. This proves that TMP functions as an intrinsic retarder whose mechanism differs from that of traditional physical adsorption or complexation-type retarders. By participating in and regulating the supply rate of the reactant (Mg^2+^), TMP achieves precise control over the hydration process, thereby establishing a solid mechanistic foundation for its application as a biocompatible retarder.

### 3.3. Compressive Strength

**Figure 7:**
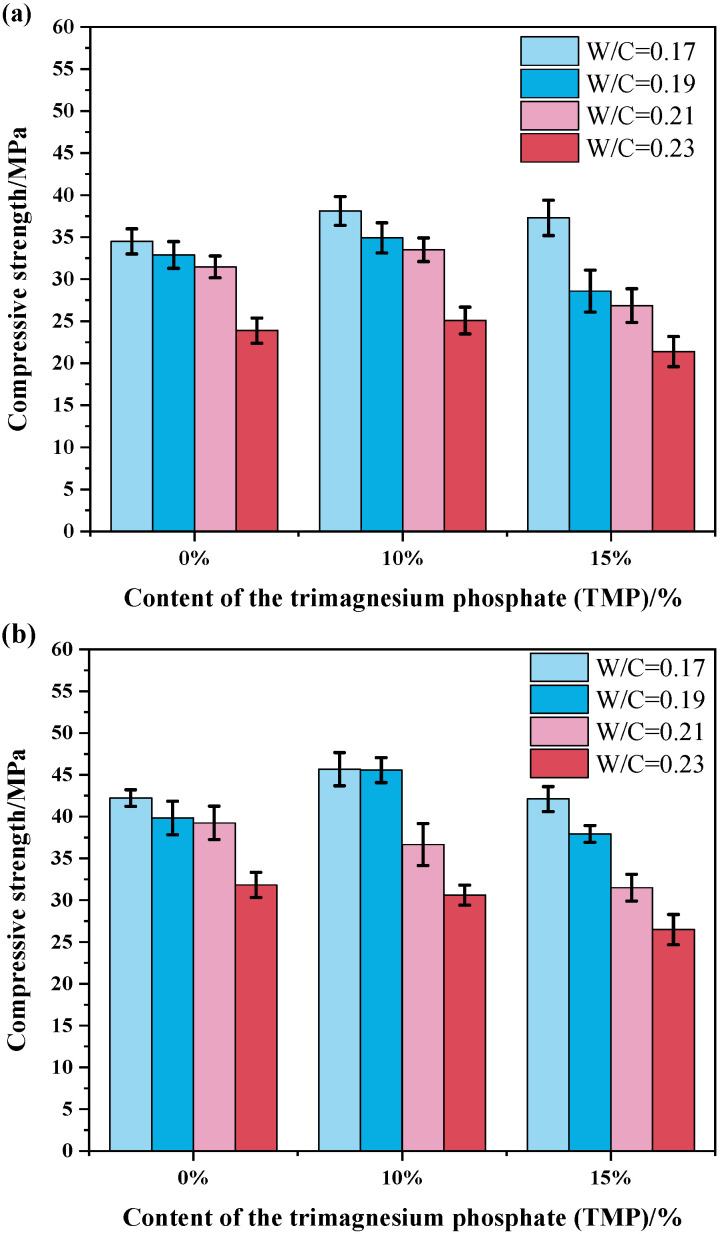
Compressive strength of the MKPC with TMP at variable W/C ratios (M/P = 4): (**a**) 1 day and (**b**) 7 days.

Figure 7 illustrates the regulating effect of TMP content on the compressive strength of MKPC at 1 day and 7 days under different water-to-cement (W/C) ratios. The results show that the incorporation of TMP exerts a non-monotonic influence on compressive strength, with its effect strongly dependent on dosage, W/C ratio, and curing age. This suggests the dual role of TMP in the system, functioning simultaneously as both a “retarder” and a “reactive magnesium source.”

As shown in Figure 7a, a TMP content of 10% represents a key optimization point for enhancing the early-age (1-day) strength of MKPC. At this dosage, the 1-day compressive strength generally increases by approximately 3–5 MPa compared to the reference group (TMP = 0%)—for example, reaching 38 MPa at W/C = 0.17. This improvement can be attributed to the retarding effect of TMP (as indicated by the previous hydration heat analysis), which likely promotes the formation of a more ordered and denser K-struvite microstructure. The moderate slowing of the reaction rate reduces micro-defects caused by rapid heat release and allows for more sufficient ion migration and crystal growth. However, when the TMP content increases to 15%, the 1-day strength decreases. This indicates that excessive retardation becomes dominant, significantly delaying the hydration process and resulting in insufficient hydration at 1 day, which prevents the formation of an effective strength framework. Moreover, the negative impact of increasing W/C on early-age strength remains valid in the TMP-containing system, consistent with the behavior of plain MKPC. This suggests that the dilution effect of water is a more fundamental factor overriding the influence of the retarder. Compressive strength values are expressed as mean ± SD (*n* = 6), as presented in Figure 7. For the 1-day strength (Figure 7a), one-way ANOVA revealed that the addition of 10% TMP significantly increased compressive strength compared to the reference group at W/C ratios of 0.17, 0.19, and 0.21 (*p* < 0.05 for all), with the most pronounced enhancement observed at W/C = 0.17 (38.1 ± 1.7 MPa vs. 33.2 ± 1.4 MPa for the reference group, *p* < 0.002). In contrast, the 15% TMP group exhibited significantly lower strength than the 10% TMP group at all W/C ratios (*p* < 0.05) and was comparable to or lower than the reference group.

At later ages (Figure 7b), the strength development pattern becomes more complex. Under low W/C conditions (≤0.19), 10% TMP still reliably enhances the 7-day strength, demonstrating that the microstructural optimization benefits conferred by retardation are sustained and even reinforced over time. However, under higher W/C conditions (≥0.21), the strengthening effect of TMP diminishes or even becomes negative. Moreover, the strength of the 15% TMP group is consistently lower than or equal to that of the reference group across all W/C ratios. This reveals a critical balance: in water-rich environments, the retarding effect of TMP may excessively delay product precipitation and densification, and coupled with its inherently low reactivity, this leads to insufficient driving force for later-age strength development. Therefore, to ensure excellent long-term mechanical performance of MKPC, especially under variable or relatively high W/C conditions, the TMP dosage should be carefully controlled—preferably not exceeding 10%, with 10% being the optimal recommended content. At 7 days (Figure 7b), the 10% TMP group maintained significantly higher strength than the reference group under low W/C conditions (W/C ≤ 0.19, *p* < 0.05), whereas no significant difference was observed at higher W/C ratios (W/C ≥ 0.21, *p* < 0.05). The 15% TMP group consistently showed lower strength than the reference group across all W/C ratios, with the difference being statistically significant at W/C = 0.17 and 0.19 (*p* < 0.05).

The evolution of strength directly reflects how TMP modulates hydration kinetics in terms of macroscopic mechanical performance. A TMP content of 10% achieves an exquisite compromise: it provides sufficient retardation to improve workability and early-age microstructure without excessively sacrificing the reaction driving force, thereby allowing later-age strength to develop fully. This finding holds significant practical implications: by precisely controlling the TMP dosage at 10%, the workable time can be effectively extended (meeting the surgical window) while ensuring that the material possesses excellent early and long-term load-bearing capacity, fully satisfying the stringent mechanical requirements for bone repair materials. Conversely, an excessively high dosage (e.g., 15%) may lead to over-retardation and impair the final mechanical performance, particularly under unfavorable mix proportion conditions.

**Figure 8:**
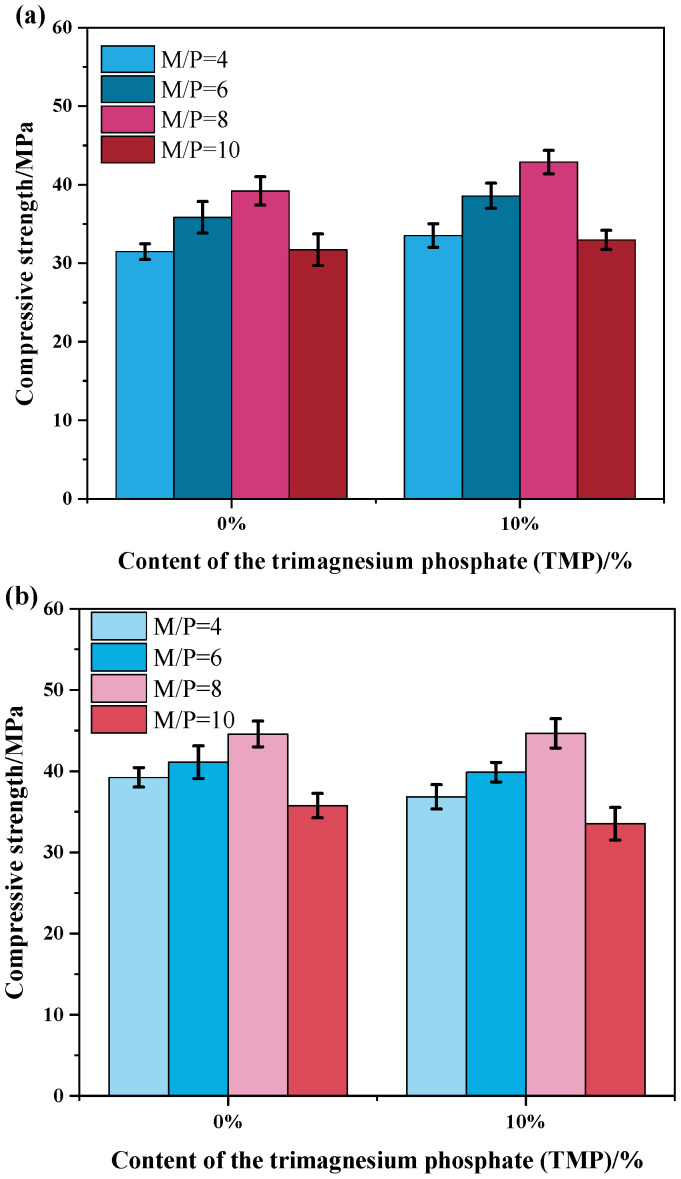
Compressive strength of the MKPC with TMP at variable M/P ratios (W/C = 0.21): (**a**) curing for 1 day and (**b**) curing for 7 days.

To systematically elucidate the effect of TMP under different chemical environments, Figure 8 further presents the combined influence of varying M/P ratios on the early (1-day) and later (7-day) compressive strength of TMP-modified MKPC at a fixed water-to-cement ratio (W/C = 0.21). As shown in Figure 8, for all series (with or without TMP), compressive strength exhibits a non-monotonic trend with increasing M/P ratio—first rising and then declining, peaking within the M/P range of 6 to 8. This pattern is fully consistent with the findings reported by Xu et al. [33]. The underlying mechanism lies in the fact that as the M/P ratio increases from a lower value (e.g., 4) to the optimal range (6–8), the relatively abundant MgO in the system not only acts as a reactant but also serves as micro-aggregates and a physical skeleton in its unreacted form, effectively enhancing the compactness and load-bearing capacity of the matrix. However, when the M/P ratio continues to rise (e.g., to 10), the relative deficiency of phosphate (KH_2_PO_4_) becomes the limiting factor, reducing the total amount of hydration product (K-struvite) that constitutes the main strength contributor. The excess MgO, lacking effective bonding, may then act as structural weak points, leading to a decline in strength.

The introduction of TMP does not alter this fundamental trend but significantly affects the absolute strength values and the progression of strength development. Firstly, within the optimal M/P range (6–8), a TMP content of 10% demonstrates a clear strengthening effect at both 1-day and 7-day ages, with strengths generally higher than those of the reference group (TMP = 0%) under the same conditions. This further confirms the earlier conclusion that 10% TMP achieves the optimal balance between retardation and reactive contribution. Its retarding action promotes the formation of a denser microstructure (e.g., by reducing microcracks), while its sustained dissolution as a supplementary magnesium source may contribute to the formation of the cementitious phase at later ages. Secondly, the incorporation of TMP alters the system’s sensitivity to changes in the M/P ratio. Compared with the reference group, TMP-containing samples exhibit a more gradual decline in strength at high M/P ratios (e.g., 10). This may be because, at high M/P ratios, the excessive highly reactive MgO in the reference group exacerbates the negative effects of early intense reactions (e.g., thermal stress-induced microcracks). In contrast, partial replacement of MgO with TMP reduces the overall reaction intensity of the system, thereby somewhat mitigating the structural deterioration caused by the imbalanced mix (phosphate deficiency).

### 3.4. Hydration Products

**Figure 9:**
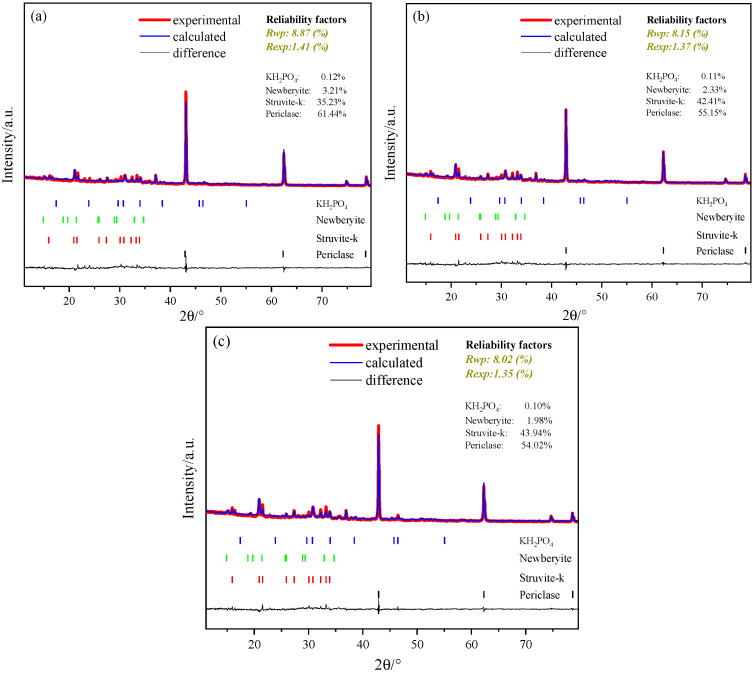
XRD Rietveld results of the magnesium phosphate cement with different amounts of TMP at 1 day: (**a**) without TMP, (**b**) 10% TMP and (**c**) 15% TMP.

To reveal the fundamental reason behind the influence of TMP on the macroscopic properties of MKPC from the perspective of phase composition, X-ray diffraction (XRD) combined with Rietveld whole-pattern fitting was employed to quantitatively analyze the phase composition of samples at 1-day age, as shown in Figure 9. The Rietveld quantitative results indicate that, regardless of TMP incorporation, the primary crystalline hydration product in all samples is K-struvite (MgKPO_4_·6H_2_O), accompanied by a small amount of newberyite (MgHPO_4_·3H_2_O), a large quantity of unreacted magnesium oxide (MgO), and trace amounts of potassium dihydrogen phosphate (KH_2_PO_4_). This key finding indicates that the introduction of TMP does not alter the final type of hydration product in the MKPC system. From the perspective of biomaterial applications, this means that TMP, as a modifier, does not compromise the inherent advantage of MKPC in forming biocompatible K-struvite as its main product, thereby providing an essential phase-chemical foundation for its safe use in biomedical scenarios such as bone repair.

The Rietveld refinement quality indicators, including Rwp, Rexp, and goodness-of-fit (χ^2^ = (Rwp/Rexp)^2^), were monitored for all samples. The χ^2^ values were consistently below 1.5, indicating satisfactory refinement quality. Given the typical uncertainty in quantitative phase analysis (approximately ±1–2% for major phases and ±2–3% for minor phases in well-refined patterns), differences in phase content of less than approximately 2% should be interpreted with caution.

Moreover, the quantitative analysis clearly reveals the regulatory effect of TMP content on the formation of key hydration products. Compared with the reference sample (TMP = 0%), the incorporation of 10% TMP significantly increases the content of K-struvite from 35.23% to 42.41%. This change in phase composition is consistent with the enhancement of macroscopic mechanical properties (see Figure 7a), as K-struvite is the primary cementitious phase and strength contributor in MKPC. The increase in its formation indicates a more developed and continuous cementitious matrix, suggesting a contribution to the improvement in early-age compressive strength at the 10% TMP dosage. However, when the TMP content is further increased from 10% to 15%, the K-struvite content only rises slightly from 42.41% to 43.94%. This difference of approximately 1.5% falls within the typical uncertainty range of Rietveld quantification, suggesting that the increase may not be statistically significant. This quantitative result is fully consistent with the macroscopic strength development trend (Figure 7a, where strength growth stagnates or declines at 15% dosage). It indicates that beyond 10%, the marginal benefit of TMP in promoting early-age hydration diminishes sharply. Excessive TMP, due to its inherently low reactivity, may act more as an inert filler in the early stage rather than effectively participating in the formation of the cementitious phase. This could lead to limited improvement in matrix densification or even hinder strength development due to dilution effects. In summary, the XRD/Rietveld quantitative analysis elucidates the “dual nature” of TMP at the phase level: at dosages ≤ 10%, TMP dissolves and participates in the reaction, optimizing the hydration process and promoting the formation of more primary cementitious phase (K-struvite), which is the fundamental reason for its enhancement of early-age strength. However, beyond this optimal dosage, its effect on promoting product formation plateaus, while its retarding and diluting negative effects become dominant, causing the strength improvement to diminish or even reverse. This finding provides critical phase-chemical evidence for determining the optimal TMP dosage (10%) in MKPC and reinforces its potential to enhance material performance while maintaining the core advantage of biocompatibility.

**Figure 10:**
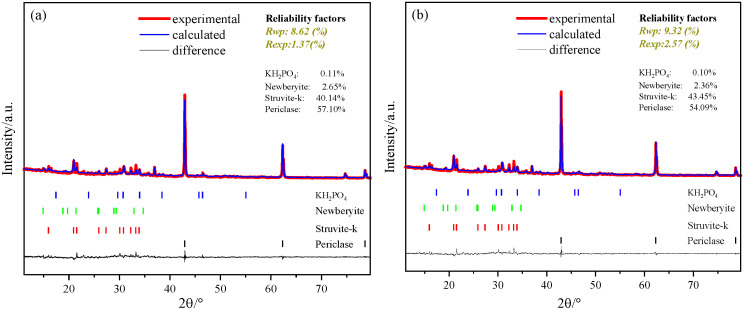

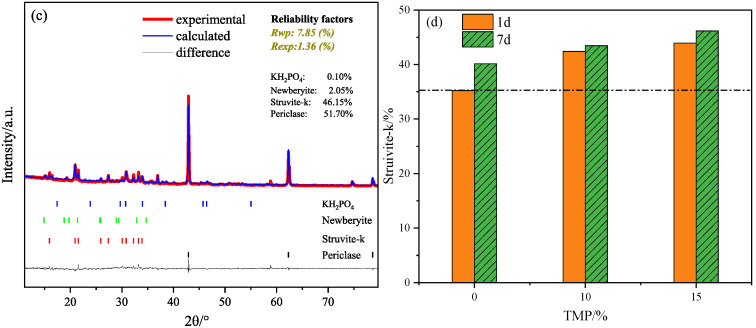
XRD Rietveld results of the magnesium phosphate cement with different amounts of TMP at 7 days: (**a**) without TMP, (**b**) 10% TMP, (**c**) 15% TMP and (**d**) formation quantity of K-struvite at 1 day and 7 days.

In addition to K-struvite, the Rietveld quantification reveals that a substantial amount of unreacted MgO remains in all formulations, ranging from approximately 61% to 54% at 1 day and from 57% to 52% at 7 days (Figure 9 and Figure 10). The presence of unreacted MgO is a well-recognized characteristic of MKPC systems, particularly when the M/P molar ratio exceeds the stoichiometric value [33]. From a mechanical perspective, unreacted MgO particles can serve as micro-aggregates or fillers within the cement matrix, potentially contributing to structural integrity and load-bearing capacity [18]; however, excessive unreacted MgO may act as weak interfaces or stress concentrators if not adequately embedded in the hydration product network [20], which partially explains the strength plateau or decline observed at higher M/P ratios (Figure 8). Regarding biocompatibility, MgO undergoes slow hydrolysis under physiological conditions, releasing Mg^2+^ and inducing local pH changes. The partial replacement of MgO with TMP reduces the content of highly reactive MgO, thereby moderating potential pH fluctuations and providing a more controlled ion release profile, which is favorable for bone repair applications [28]. Thus, the balance between sufficient unreacted MgO for mechanical support and limited residual MgO to avoid adverse biological effects further supports the suitability of TMP-modified MKPC as a bone repair material.

To elucidate the long-term hydration process of MKPC influenced by TMP, Figure 10 presents the XRD/Rietveld quantitative analysis results of samples at 7 days, along with a comparison with the 1-day data (Figure 9) in Figure 10d. This analysis aims to reveal the evolution of hydration products over time, thereby explaining the underlying reasons for macroscopic strength development. The phase analysis at 7 days (Figure 10a–c) confirms that, regardless of TMP incorporation, the primary crystalline hydration product in the system remains K-struvite, with no detection of new crystalline phases. This result is consistent with the conclusion drawn at 1 day, providing strong evidence once again that TMP does not alter the final phase composition of MKPC. From the perspective of long-term material safety, this ensures that the modified MKPC can maintain its inherent, biocompatible chemical stability in vivo, avoiding biological risks associated with the formation of unknown or harmful by-products—a core prerequisite for its application as a biomaterial.

Key insights into the hydration process are embedded in the time-evolution data of K-struvite content (Figure 10d). For the reference group (TMP = 0%), the K-struvite content increases from 35.23% at 1 day to 40.14% at 7 days, representing a growth of approximately 5%, indicating that hydration continues after 1 day. This aligns with the conventional understanding that MKPC exhibits ongoing strength development at later ages.

However, the introduction of TMP significantly alters this hydration kinetic pattern. At a TMP content of 10%, the K-struvite content increases only marginally from 42.41% at 1 day to 43.45% at 7 days, a growth of less than 1%. This quantitative data clearly demonstrates that the core cementitious reaction in TMP-modified MKPC is largely completed during the early stage (within 1 day). This is consistent with the compressive strength development pattern described earlier (Figure 7): TMP-modified samples already achieve a high strength level at 1 day, while the strength gain at 7 days is relatively limited. This pattern of “rapid early-age hydration followed by a plateau at later ages” directly reflects the dual role of TMP as both a slow-release magnesium source and a retarder. Its retarding effect does not indefinitely delay the reaction but rather optimizes the early-age reaction pathway, promoting more efficient and concentrated formation of K-struvite. Once the dense microstructure is formed at early ages, restricting further transport of water and ions, the later-age reaction naturally slows down.

**Figure 11:**
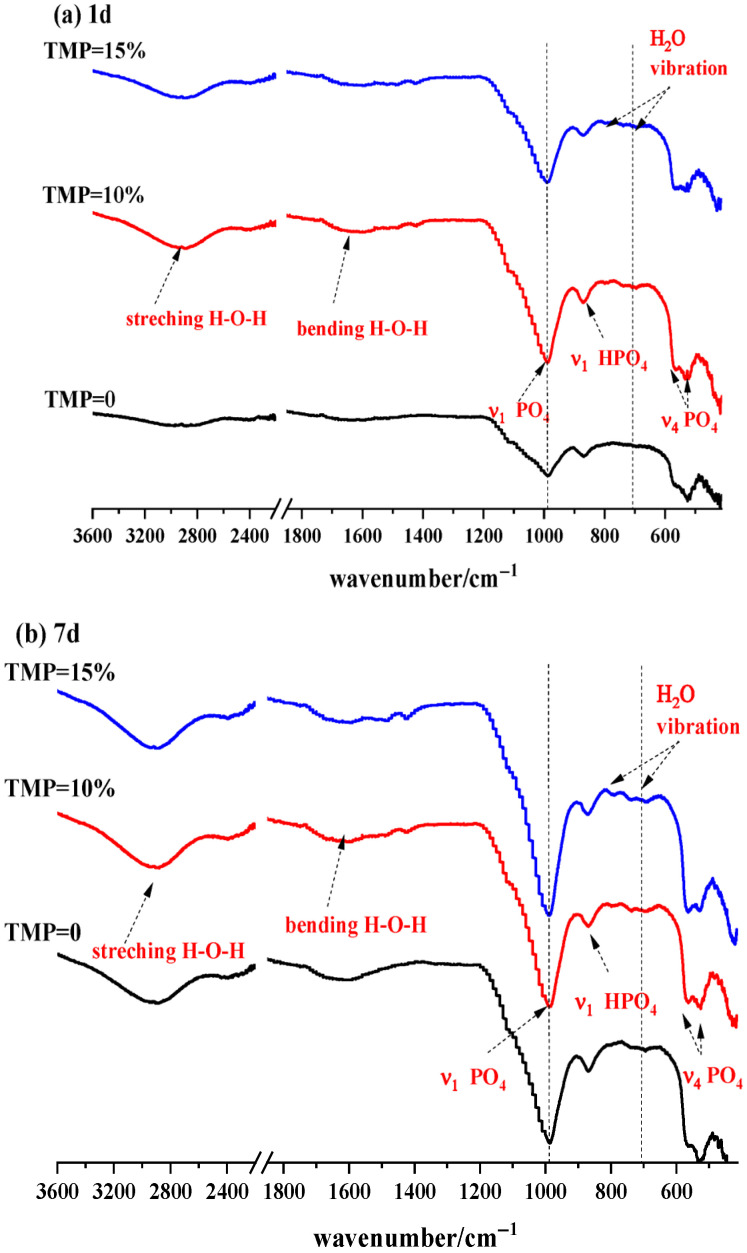
FTIR spectra of MKPC with TMP at (**a**) 1 day and (**b**) 7 days.

FTIR spectra (Figure 11) provide critical information for further revealing the influence of TMP on the chemical bonding state and amorphous microstructure of MKPC hydration products, complementing the phase-quantitative results from XRD and collectively constructing a comprehensive hydration picture spanning from crystalline to amorphous phases. The spectra of all samples at 1-day and 7-day ages exhibit characteristic absorptions of the MKPC system (Figure 11a,b). First, the sharp peaks located at ~1000 cm^−1^ and ~560 cm^−1^ are attributed to the ν_1_ symmetric stretching vibration and ν_4_ bending vibration of the PO_4_^3−^ group in K-struvite (MgKPO_4_·6H_2_O), respectively, while the shoulder at ~900 cm^−1^ corresponds to the ν_1_ vibration of HPO_4_^2−^ in newberyite (MgHPO_4_·3H_2_O) or unreacted intermediates. These characteristic peaks are highly consistent with the XRD results (Figure 9 and Figure 10), jointly confirming the main composition of the crystalline phases.

More importantly, FTIR clearly reveals structural information on the amorphous phases that cannot be directly detected by XRD. The exceptionally broad and strong absorption band extending from ~3400 cm^−1^ to ~2400 cm^−1^ in the spectra provides clear evidence for the presence of strongly bound water molecules and ultrafine/amorphous hydration phases in the system. Within this continuous band, the two broad peaks at ~2900 cm^−1^ and ~2400 cm^−1^ are distinctive features, with the latter particularly indicating the formation of very strong hydrogen bonds or “short strong hydrogen bonds.” This unique hydrogen-bonding network commonly exists in complex amorphous phases composed of ultrafine particles, amorphous magnesium phosphate gels, and structurally highly disordered bound water. Such phases constitute an important component of the MKPC microstructure and likely contribute significantly to early setting, micro-densification, and final mechanical performance of the material.

By comparing the spectra at 1 day and 7 days (Figure 11a vs. Figure 11b), the kinetics of hydration structure evolution under the influence of TMP can be traced: the peak intensity at ~1000 cm^−1^ (K-struvite) increases for all samples at 7 days, indicating continued growth of the crystalline phase. However, the extent of this increase is notably smaller in TMP-containing samples. This observation fully aligns with the quantitative XRD/Rietveld data: the K-struvite content in the TMP-free sample increased by about 5% from 1 day to 7 days, while that in the 10% TMP sample increased by only about 1% (Figure 10d). This directly confirms, at the chemical bonding level, the earlier conclusion that TMP incorporation concentrates K-struvite formation primarily in the early hydration stage, with later crystallization growth tending to plateau. Simultaneously, the relative weakening of the peak intensity at ~900 cm^−1^ (assigned to HPO_4_^2−^) with age indicates the ongoing conversion of acidic phosphate intermediates into the more stable K-struvite. The FTIR analysis reveals the dual nature of TMP’s role at the molecular vibration level: on one hand, it does not alter the final chemical product type of the system (predominantly K-struvite); on the other hand, by acting as a slow-release magnesium source, it significantly regulates the hydration kinetics, shifting the crystallization process of struvite forward and essentially completing it early. This characteristic of “early rapid structuring” corresponds perfectly with the material’s excellent early-age strength (Figure 7) and setting behavior suitable for rapid construction/repair (Figure 1 and Figure 2). Furthermore, the presence of strongly bound water and amorphous phases revealed by FTIR may be another key factor contributing to the denser microstructure—and consequently higher early strength—of TMP-modified material.

**Figure 12:**
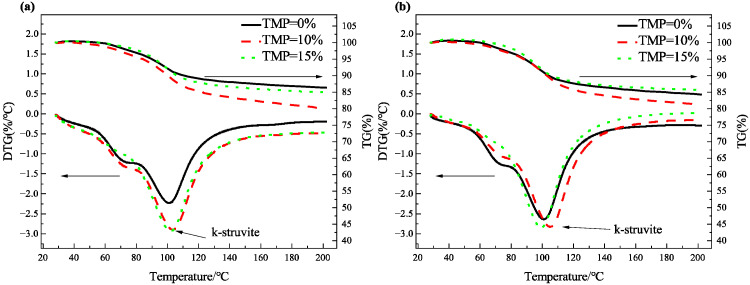
TG-DTG of MKPC with TMP at (**a**) 1 day and (**b**) 7 days.

Figure 12 presents the TG-DTG curves of hydration products for MKPC with TMP at 1 day (Figure 12a) and 7 days (Figure 12b). As observed in Figure 12, two weak weight-loss peaks appear in the temperature range of 60–90 °C. Combined with the XRD results (Figure 9 and Figure 10), these peaks likely correspond to newberyite formed during the early hydration of MKPC, while the possibility of certain amorphous phases or gel phases containing loosely bound water cannot be excluded—potentially including an intermediate adsorbed-water-bearing phase such as Mg_3_(PO_4_)_2_·xH_2_O, which may form as an amorphous precursor before the development of highly crystalline K-struvite. The strong weight-loss peak in the temperature range of 90–110 °C is primarily attributed to K-struvite, a result consistent with the XRD findings. Moreover, at 1 day of hydration, the K-struvite weight-loss peak intensifies with increasing TMP content, indicating that TMP incorporation significantly enhances the formation of K-struvite in MKPC. This observation aligns with the XRD-Rietveld quantitative analysis shown in Figure 9. However, at 7 days of hydration, the effect of TMP on the K-struvite weight-loss peak becomes less pronounced. Notably, when the TMP content exceeds 10%, the intensity of the K-struvite weight-loss peak decreases markedly, suggesting that excessive TMP incorporation hinders the further formation of K-struvite.

### 3.5. Microstructures

**Figure 13:**
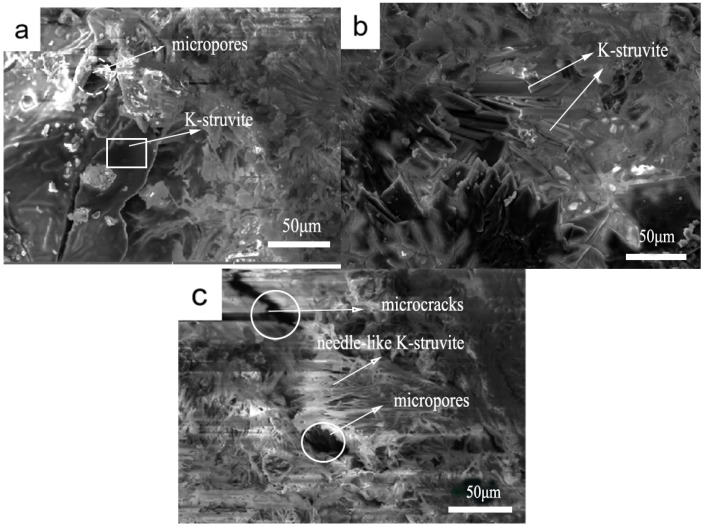
Microscopic structure of MKPC with TMP at 7 days (M/P = 4, W/C = 0.21): (**a**) TMP = 0, (**b**) TMP = 10% and (**c**) TMP = 15%.

Figure 13 presents the typical micro-morphologies of MKPC specimens incorporating different proportions of trimagnesium phosphate (TMP) after 7 days of hydration. In the absence of TMP (Figure 13a), the microstructure consists of loosely interwoven, irregularly sized flaky and blocky K-struvite crystals, with evident micropores visible in the matrix. With the incorporation of 10% TMP (Figure 13b), the morphology undergoes a significant transformation: K-struvite crystals appear as uniform, fine, short-columnar or needle-like particles that closely interlock to form a continuous, dense matrix with low porosity, while unreacted particles are substantially reduced. However, when the TMP content increases to 15% (Figure 13c), structural homogeneity declines, manifesting as localized needle-like crystal formation, microcracks, and flocculent aggregates.

This systematic evolution in morphology is highly consistent with the phase and performance data obtained in this study. The uniform and dense structure formed at 10% TMP content provides direct micro-scale evidence for the highest K-struvite content (42.41% at 1 day) and optimal early-age compressive strength observed in XRD quantitative analysis. TMP particles act as additional nucleation sites, promoting abundant and uniform nucleation of K-struvite, thereby refining the crystals and optimizing matrix packing. In contrast, the porous structure and substantial amount of unreacted MgO in the TMP-free sample correspond to its lower early-age strength and degree of reaction. The structural inhomogeneity observed at 15% TMP content explains microscopically why its compressive strength is lower than that of the 10% group: excessive TMP may disrupt the uniform precipitation of hydration products or induce localized micro-stress concentration, creating weak interfaces. In summary, SEM observations confirm that an optimal TMP dosage exists (10% in this study), which enables the achievement of maximally densified microstructure in MKPC, thereby optimizing its macroscopic mechanical performance.

## 4. Discussion

The present study demonstrates that trimagnesium phosphate (TMP) serves as an effective and biocompatible retarder for magnesium potassium phosphate cement (MKPC), successfully addressing the critical challenge of excessively rapid setting while enhancing early mechanical properties. The following discussion synthesizes the key findings and their implications:

### 4.1. Dual-Functionality of TMP: Simultaneous Retardation and Strength Enhancement

**Figure 14:**
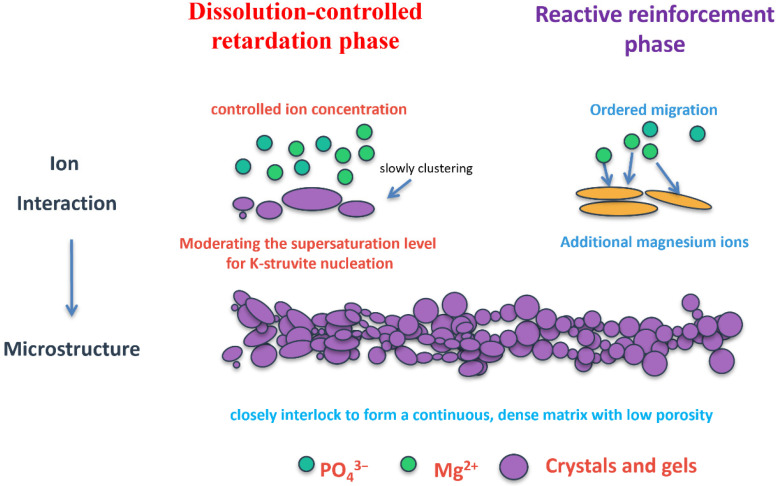
Proposed theoretical model of TMP’s dual-function mechanism based on the experimental observations.

A significant advancement revealed in this study is the dual functionality of TMP, which distinguishes it from conventional chemical retarders. While typical retarders for MKPC (e.g., borax, citric acid) often exhibit a trade-off between setting time extension and mechanical property reduction, TMP achieves both objectives simultaneously. The extension of setting time from 9–13 min to 10–19 min could provide a working window potentially suitable for surgical procedures, pending validation under relevant handling conditions, while the enhancement of early compressive strength (1-day: 35.2 MPa with 10% TMP vs. 28.5 MPa for control) addresses the need for immediate mechanical support in orthopedic applications. Based on the calorimetry, XRD, and microstructural observations, a theoretical model is proposed to describe TMP’s dual-function mechanism (Figure 14): (1) Dissolution-controlled retardation phase: TMP’s low solubility is hypothesized to create a sustained-release reservoir of Mg^2+^ and PO_4_^3−^ ions, moderating the supersaturation level for K-struvite nucleation. This contrasts with conventional retarders that typically adsorb on MgO surfaces or form complexes with reaction intermediates. (2) Reactive reinforcement phase: As TMP gradually dissolves, it is hypothesized to provide additional magnesium ions that participate in K-struvite formation, contributing to matrix densification rather than acting as inert filler. This model provides a plausible interpretation of the experimental observations, although direct evidence (e.g., in situ Mg^2+^ concentration monitoring, nucleation site characterization) remains to be obtained in future studies.

This dual functionality stems from TMP’s unique retardation mechanism. Unlike chemical retarders that function through surface adsorption or complexation, TMP operates as a “reactive retarder” by partially replacing highly reactive dead-burned MgO with a less soluble magnesium source. The slower dissolution kinetics of TMP moderate Mg^2+^ release, thereby delaying K-struvite formation without introducing foreign chemical species. Calorimetry results are consistent with this controlled hydration kinetics, while SEM observations reveal that 10% TMP promotes the formation of uniform, fine needle-like K-struvite crystals that interlock to create a dense, low-porosity matrix. This microstructural refinement, combined with accelerated early K-struvite formation (42.41% at 1 day vs. 35.23% for control), explains the concurrent setting time extension and strength enhancement. Notably, TMP has been reported to exhibit favorable biocompatibility and degradability [28,31], further supporting its potential for biomedical applications.

It should be noted that the proposed mechanism is primarily supported by indirect evidence from calorimetry, phase quantification, and microstructural characterization. Direct experimental verification, such as in situ monitoring of Mg^2+^ release kinetics or direct observation of nucleation site formation, would be required to fully validate the proposed model.

### 4.2. Optimal Performance at 10% TMP Replacement: Balancing Multiple Properties

The identification of 10% TMP as the optimal replacement level represents a critical finding with practical significance. This dosage achieves the best balance among three key performance parameters: (1) setting time extension to clinically suitable ranges (10–19 min), (2) enhancement of early mechanical properties (35.2 MPa at 1 day), and (3) microstructural densification with refined crystal morphology. The performance deterioration observed at 15% TMP content highlights the importance of precise dosage control and suggests that excessive TMP may lead to structural heterogeneity, microcrack formation, and reduced strength due to dilution effects and disrupted hydration kinetics. The optimal performance at 10% TMP is further supported by the minimal impact on fluidity, indicating maintained injectability and workability for minimally invasive surgical techniques. Additionally, FTIR analysis confirms that TMP does not alter the fundamental chemical composition of hydration products, preserving the essential chemical features required for biocompatibility. This optimal formulation offers a combination of extended working time, enhanced early strength, and literature-supported biocompatibility—three characteristics that are essential for successful bone repair applications.

### 4.3. Potential for Biomedical Applications and Future Perspectives

It should be emphasized that the present study focuses on the materials science aspects of TMP-modified MKPC. No biological evaluations (e.g., cytotoxicity, hemocompatibility, or in vivo responses) were conducted. Therefore, the biomedical implications discussed below are speculative and based solely on material properties and literature reports. Biological validation, particularly toxicological assessment, is an essential prerequisite before any consideration of clinical translation. The extended setting time window of 10–19 min achieved with TMP modification may help address a critical limitation of conventional MKPC if considered for biomedical use. In terms of handling characteristics, this range could provide adequate time for precise placement and shaping in surgical scenarios, although such performance remains to be validated under actual operating conditions. The enhanced early strength (35.2 MPa) exceeds the typical requirements for cancellous bone replacement (2–10 MPa) and falls within the lower range of cortical bone strength (100–150 MPa), suggesting potential suitability for load-bearing applications pending further mechanical and biological validation. From a biocompatibility perspective, TMP is considered to offer advantages over conventional chemical retarders based on its known composition and previous reports [28,31], as it has been reported to be biocompatible and biodegradable, potentially eliminating concerns regarding cytotoxicity from leachable chemical additives. These characteristics suggest that TMP-modified MKPC may be suitable for bone repair applications where long-term material safety is a key consideration.

It is important to acknowledge that the presence of unreacted MgO, which remains abundant in all formulations (Figure 9 and Figure 10), may undergo slow hydrolysis under physiological conditions, leading to local pH elevation over time. While moderate alkalinity can be beneficial for osteoblast activity, excessive or sustained pH increases may provoke inflammatory responses or impair cell viability [28]. In this study, the partial replacement of MgO with TMP reduces the overall content of highly reactive MgO, thereby potentially moderating the extent and duration of pH elevation compared to conventional MKPC. Moreover, TMP itself has been reported to exhibit neutral to mildly basic behavior and good cytocompatibility [28,31]. Nevertheless, the present study did not include direct pH measurements of hardened cements exposed to simulated body fluids over extended periods. Systematic evaluation of pH evolution under physiological conditions, along with in vitro cytocompatibility testing, is therefore a critical next step to fully establish the safety profile of TMP-modified MKPC for bone repair applications.

Future research should focus on three key areas to further evaluate the biomedical potential of TMP-modified MKPC: (1) comprehensive biological evaluation, including cytotoxicity testing, hemocompatibility assessment, and in vitro osteogenic differentiation assays, to establish the safety profile of the material; (2) long-term stability assessment under simulated physiological conditions; and (3) comparative studies with other biocompatible retarders to establish relative advantages. Toxicological evaluation is a critical next step, as the present study provides only the materials science foundation. These investigations are essential to determine whether the promising material properties observed can ultimately support biomedical applications.

## 5. Conclusions

This study systematically investigates the feasibility of using trimagnesium phosphate (TMP) as a biocompatible retarder for magnesium potassium phosphate cement (MKPC). The comprehensive evaluation of TMP’s effects on setting behavior, mechanical properties, hydration kinetics, and microstructure reveals its unique dual-function characteristics. The main findings are summarized as follows:

(1) TMP successfully extends the setting time of MKPC from 9–13 min (without TMP) to 10–19 min. This extension is achieved without compromising the fundamental acid-base reaction mechanism of MKPC. The retardation effect is attributed to TMP’s slower dissolution rate compared to dead-burned MgO, which moderates the release of Mg^2+^ ions and delays K-struvite nucleation and growth.

(2) Contrary to conventional retarders that typically compromise mechanical strength, 10% TMP incorporation improves early compressive strength. The 1-day compressive strength reaches 35.2 MPa with 10% TMP, representing a 23.5% increase compared to the control group (28.5 MPa). This unique combination of setting time extension and strength enhancement distinguishes TMP from typical chemical retarders and offers a potential solution to a critical challenge in MKPC development.

(3) Microstructural analysis confirms that TMP promotes the formation of denser, more uniform K-struvite crystals with improved interlocking. The refined microstructure contributes to enhanced mechanical performance and reduced porosity. TMP participates in the hydration reaction rather than acting as an inert filler, contributing to matrix densification through its gradual dissolution and reaction.

(4) The retarding mechanism involves TMP’s controlled dissolution, regulating the supersaturation level for K-struvite formation, while its participation in the hydration reaction contributes to strength development. This dual-function mechanism—simultaneously acting as a dissolution-controlled retarder and a reactive magnesium source—represents a novel approach to MKPC modification.

It should be noted that the present study is limited to materials characterization. While the results are promising, further biological validation—particularly toxicological assessment—is required before any consideration of biomedical applications.

## Figures and Tables

**Table 1:** Mix proportion of MKPC.

NO.	M/P	W/C	TMP
1	4	0.21	10%
2	6	0.21	10%
3	8	0.21	10%
4	10	0.21	10%
5	4	0.17	10%
6	4	0.19	10%
7	4	0.23	10%
8	4	0.21	0
9	4	0.21	15%

## Data Availability

The original contributions presented in this study are included in the article. Further inquiries can be directed to the corresponding author.
